# Population Structure of *Montastraea cavernosa* on Shallow versus Mesophotic Reefs in Bermuda

**DOI:** 10.1371/journal.pone.0142427

**Published:** 2015-11-06

**Authors:** Gretchen Goodbody-Gringley, Chiara Marchini, Alex D. Chequer, Stefano Goffredo

**Affiliations:** 1 Bermuda Institute of Ocean Sciences, 17 Biological Lane, St. Georges, Bermuda GE 01; 2 Marine Science Group, Department of Biological, Geological and Environmental Sciences, University of Bologna, Via F. Selmi 3, 40126 Bologna, Italy, European Union; 3 Ocean Support Foundation, Suite 1222, 48 Par-la-Ville Road, Hamilton, Bermuda HM 11; Biodiversity Research Center, Academia Sinica, TAIWAN

## Abstract

Mesophotic coral reef ecosystems remain largely unexplored with only limited information available on taxonomic composition, abundance and distribution. Yet, mesophotic reefs may serve as potential refugia for shallow-water species and thus understanding biodiversity, ecology and connectivity of deep reef communities is integral for resource management and conservation. The Caribbean coral, *Montastraea cavernosa*, is considered a depth generalist and is commonly found at mesophotic depths. We surveyed abundance and size-frequency of *M*. *cavernosa* populations at six shallow (10m) and six upper mesophotic (45m) sites in Bermuda and found population structure was depth dependent. The mean surface area of colonies at mesophotic sites was significantly smaller than at shallow sites, suggesting that growth rates and maximum colony surface area are limited on mesophotic reefs. Colony density was significantly higher at mesophotic sites, however, resulting in equal contributions to overall percent cover. Size-frequency distributions between shallow and mesophotic sites were also significantly different with populations at mesophotic reefs skewed towards smaller individuals. Overall, the results of this study provide valuable baseline data on population structure, which indicate that the mesophotic reefs of Bermuda support an established population of *M*. *cavernosa*.

## Introduction

In recent years, coral reefs have undergone drastic decline due to numerous anthropogenic impacts to environmental conditions including eutrophication, disease, the loss of herbivory, and bleaching associated with ocean warming [1–4]. Currently, nearly 30% of the world's coral reefs are considered severely damaged, and close to 60% are in danger of being lost by 2030 [5]. These losses are particularly pronounced on shallow water reefs of the Caribbean, where the comprehensive study by Jackson et al. [1] reports an overall decline in coral cover of 59%, from an average of 33% before 1984 to 14.3% since 2005. Deep reef systems in the mesophotic zone (>30m), however, have not experienced the same trend, displaying relatively stable coral populations over time [6]. Yet, in comparison to shallow-water coral reefs, mesophotic reefs have received little attention [7].

Mesophotic coral ecosystems (MCE’s) are comprised of a variety of taxa, including sponges, macroalgae, and azooxanthellate corals, as well as light-dependent zooxanthellate corals that exist in zones between approximately 30m and 150m in tropical and subtropical zones [8–10]. These regions tend to exist in low energy deep fore-reef zones that are characterized by steep gradients in light and temperature [8]. Typically the depth at which light is reduced to 1% of the available surface light defines the lower limits of the mesophotic zone [11]. Previous technological limitations have presented major challenges to conducting research on MCE’s, resulting in limited understanding of the bathymetric and geographic extent of MCE’s and the biodiversity and community structure they support across regions. Even basic taxonomic and systematic characterization of these communities is unknown, underscoring the importance of establishing baseline information on species assemblages and the roles they play in ecosystem function [10, 12].

Analyses of size-frequency distributions can reveal characteristics of species populations as they represent stages of population growth and decline [13]. Population size structure results from variations in rates of colony growth, recruitment and mortality, and may indicate individual sensitivities to life-history processes and environmental variation. The life cycle of modular organisms such as scleractinian corals, however, is complicated by processes such as fragmentation, fission, fusion, and partial mortality, making the relationship between surface area and age difficult to interpret [14]. Yet coral colony surface area can be correlated to age if partial mortality is low, and thus characterizations of population size-frequency distributions may provide critical demographic information, particularly for massive, non-branching colonies [13, 15–19]. Describing coral populations in terms of population size-frequency, therefore, can provide a snap-shot of current reef condition and if monitored over time may serve as an indicator for stability or decline [17, 20].

The aim of this study is to provide baseline characterization of population structure for the dominant zooxanthellate coral at adjacent shallow and mesophotic reefs in Bermuda. As such, this study provides an initial assessment of mesophotic reef condition in relation to environmental conditions that vary with depth, such as temperature and nutrient levels. Fricke and Meischner [21] conducted the only comprehensive study of mesophotic reef composition in Bermuda using submersible video transect surveys. Their study found species diversity decreased drastically below 40m. Among the species found in these mesophotic zones include *Agaricia fragilis*, *Stephanocoenia michelini*, *Madracis decactis*, *Scolymia cubensis*, *Montastraea cavernosa* and *Orbicella franksii*, with *M*. *cavernosa* and *O*. *franksii* being the dominant representatives below 30m. Using *in situ* diver-led surveys we examine variations in colony density, surface area, percent cover and size-frequency distributions and provide baseline data on the population structure of *M*. *cavernosa* on mesophotic reefs in Bermuda.

## Materials and Methods

### Ethics Statement

Surveys for this study were conducted in public areas outside of any marine reserves and did not require approval or permitting. No specimens were manipulated or collected from reef sites in completing this study and care was taken to avoid contact with benthic substrata.

### Site and Species Selection

Located 32°N, 64°W, Bermuda’s sub-tropical coral reefs represent the northernmost reef system in the Atlantic. The shallow rim reefs of this pseudo-atoll encircle the platform, dropping quickly to deep mesophotic reefs. Thus, deep reefs are easily accessible in Bermuda and corals surviving in these zones are both at their latitudinal and bathymetric limits. Furthermore, shallow water coral cover in Bermuda ranks among the highest in the Caribbean with an estimated cover of 38.6% [1]. Bermuda, therefore, is an ideal and important location in which to study coral community composition and connectivity across a depth gradient.

*M*. *cavernosa* (Linnaeus, 1767) is a common reef building coral on fore reef slopes throughout the Caribbean and western Atlantic, extending from Bermuda to Brazil and the West African coast [22, 23]. *M*. *cavernosa* is considered an ‘extreme’ depth-generalist [9], as it inhabits depths from 3–100m across its geographical range [21, 24, 25]. Along its bathymetric distribution, *M*. *cavernosa* exhibits significant phenotypic plasticity in morphology, rates of respiration, and primary productivity [25–27], and is the only hermatypic species documented to survive below 70m in Bermuda [21].

### Surveys

**Fig 1:**
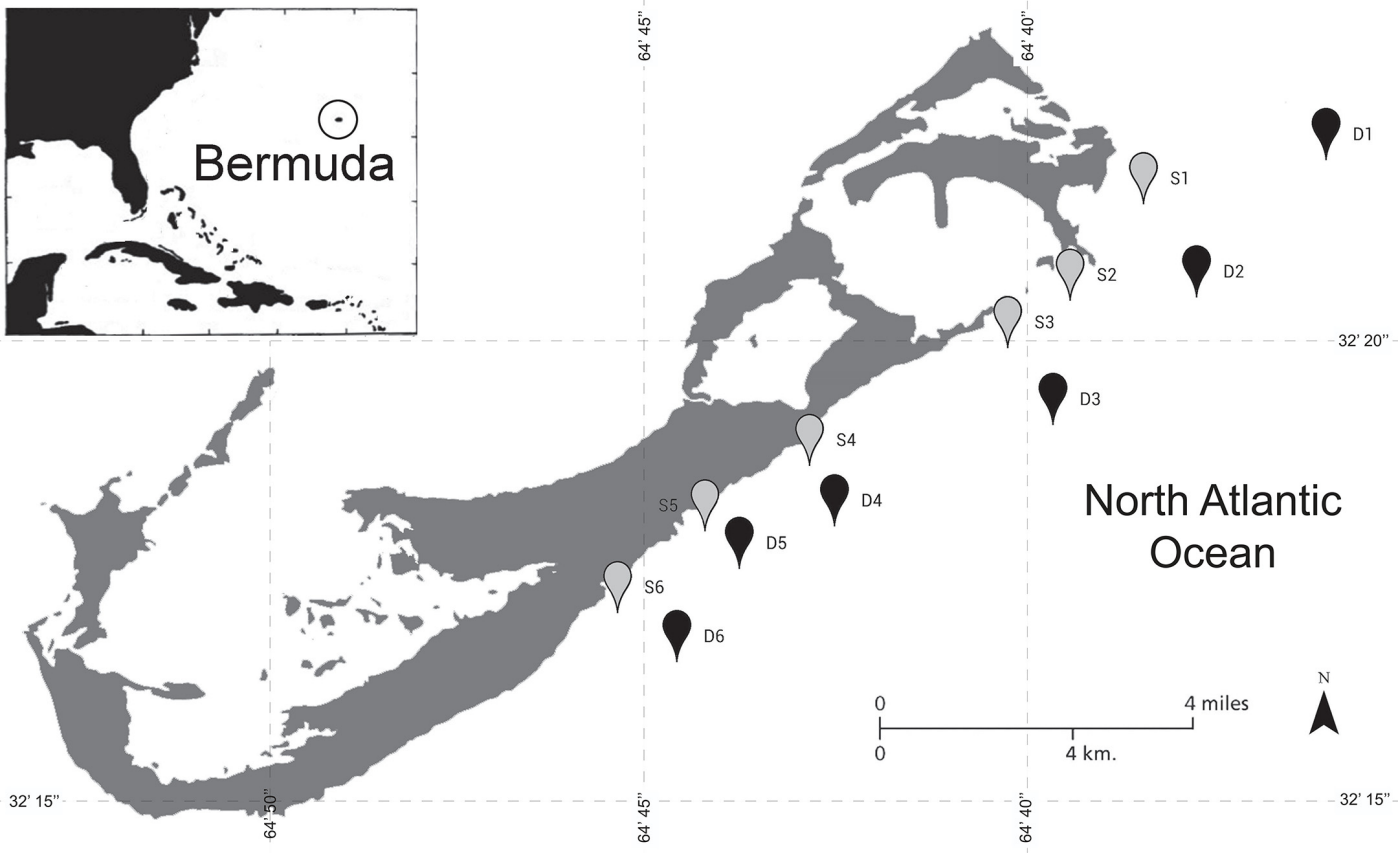
Survey Map. Survey locations on the south shore of Bermuda at shallow (10m; gray markers) and mesophotic (45m; black markers) sites. S1: Rita, 10m; D1: XL, 45m; S2: Coopers, 10m; D2: Coopers, 45m; S3: Tuckers, 10m; D3: Tuckers, 45m; S4: Spittal, 10m; D4: Spittal, 45m; S5: Devonshire, 10m; D5: Devonshire, 45m; S6: Hungry Bay, 10m; D6: Hungry Bay, 45m.

Twelve coral surveys were performed between August 17^th^ and December 28^th^ 2014 to estimate abundance and surface area of *M*. *cavernosa* colonies between shallow and mesophotic reef sites in Bermuda (Fig 1). Six surveys were conducted at shallow sites (10m depth), and six were conducted at nearby mesophotic sites (45m depth). Site names, map labels, GPS coordinates, and survey dates are included in Table 1. Site locations were selected based on accessibility and visual identification of reef structure at mesophotic sites. Paired shallow sites were selected as the nearest site encountered at 10m depth traveling up the reef slope perpendicular to the shoreline. At each site, all *M*. *cavernosa* colonies with greater than 50% of the colony located within 1m of either side of a 30m transect tape (60m^2^ total area per survey) were counted and largest surface diameter measured (to nearest cm). Diameter was chosen as a metric for ease of completing surveys at depth with minimal bottom time (maximum bottom time of 25 min.). Transects at each site were laid along the reef slope to ensure a constant depth, beginning at the closest non-living reef structure encountered upon reaching the benthos to which the tape could be secured. A colony was defined as any autonomous coral skeleton with living tissue as described by Meesters et al. [19].

**Table 1:** Survey Locations. Details of site locations surveyed including site map label (Fig 1), corresponding site name, depth (m), date surveyed, and GPS location (latitude and longitude).

Map Label	Site Name	Depth (m)	Date	Latitude	Longitude
S1	Rita	10	17-Aug-14	N32° 21' 29.3"	W64° 38' 29.3"
S2	Coopers	10	17-Aug-14	N32° 20' 28.4"	W64° 39' 28.1"
S3	Tuckers	10	5-Sep-14	N32° 19' 57.7"	W64° 40' 16.5"
S4	Spittal	10	5-Sep-14	N32° 18' 42.3"	W64° 42' 53.4"
S5	Devonshire	10	28-Dec-14	N32° 18' 0.7"	W64° 44' 16.1"
S6	Hungry Bay	10	28-Dec-14	N32° 17' 8.5"	W64° 45' 26.1"
D1	XL	45	17-Aug-14	N32° 21' 58.0"	W64° 36' 5.3"
D2	Coopers	45	17-Aug-14	N32° 20' 29.6"	W64° 37' 47.2"
D3	Tuckers	45	5-Sep-14	N32° 19' 8.8"	W64° 39' 41.0"
D4	Spittal	45	5-Sep-14	N32° 18' 3.7"	W64° 42' 34.7"
D5	Devonshire	45	21-Dec-14	N32° 17' 36.5"	W64° 43' 48.9"
D6	Hungry Bay	45	28-Dec-14	N32° 16' 37.5"	W64° 44' 39.4"

### Population Structure Analyses

Density of *M*. *cavernosa* colonies (# of colonies 60m^-2^) met the assumptions of normality and equal variance and was analyzed by depth using a Student’s *t*-test. Mean colony diameter was used to calculate surface area of each colony using the following equation: surface area = 2π(diameter/2)^2^. Colony surface area was logarithmically transformed to reduce non-normality and heteroschedasticity and for each site, geometric mean (μ), standard deviation (SD), skewness (g_1_), and kurtosis (g_2_) were calculated. Mean colony surface area, standard deviation and skewness were compared by depth using the Student’s *t*-test (n = 6), and kurtosis was compared by depth using a Mann-Whitney *U*-test [28–30]. These statistics describe the shape of a distribution and allow comparisons between populations at different depths independent of colony surface area [13, 19]. The total surface area per 60m^2^ transect was also used to calculate percent cover of *M*. *cavernosa* (% cover 60m^-2^). Data was transformed to arcsine values and compared by depth using a Student’s *t*-test.

Mean size-frequency distributions were generated for each depth zone (shallow and mesophotic) and compared with each other by a Kolmogorov-Smirnov test and to a normal distribution using a Shapiro-Wilk W test [31–33]. Additionally, size-frequency distributions within each shallow and mesophotic site were compared using a Kolmogorov-Smirnov test. Similarity of size-frequency distributions between shallow and mesophotic sites was calculated with the Spearman rank-correlation coefficient by dividing colony numbers into 10 surface area size classes based on a logarithmic scale (class borders were < 0.5, 1.0, 1.5, 2.0. 2.5, 3.0, 3.5, 4.0, 4.5, and >4.5 cm^2^). Correlation coefficients were not normally distributed, and group means were tested with the Mann-Whitney *U*-test. All analyses were computed using PASW Statistics 17.0.

Size-frequency distributions within sites were also examined with a principal coordinate ordination (PCO) analysis based on Euclidean similarity, which generates a two-dimensional plot. PCO analysis is an equivalent to principal component analysis (PCA), but with more flexibility of resemblance measures [34] and allows spatial visualization of dissimilarities among sites and between depths. This analysis was performed using PRIMER version 6.

### Nutrient and Temperature Analyses

Seawater samples were collected at two shallow sites and two mesophotic sites during survey dives (Tuckers and Spittal). Four replicate samples were collected at each site. Analysis of nitrate (NO_3_), nitrite (NO_2_), and silicate (SiO4^-2^) were conducted at BIOS with a Seal Analytical AA3 continuous flow analyzer. Concentrations of nitrogen (NO_3_ + NO_2_) and silicate at each site met the assumptions of normality and equal variance and were analyzed by depth using Student’s *t*-tests (n = 4 per site). Seawater temperature readings were recorded at each of the surveyed shallow and mesophotic sites between July 2014 and January 2015 using a Shearwater Petrel dive computer. Each site was visited twice during this time period for a total of 12 paired temperature readings. Mean temperatures were compared by depth using a Student’s *t*-test (n = 6).

## Results

### Distribution Parameters

Table 2 gives the geometric mean surface area, skewness, kurtosis, maximum colony surface area, standard deviation, the probability that the sample is from a normal distribution, and the sample size at each site. Each parameter is also given for all shallow sites and all mesophotic sites combined.

**Table 2:** Distribution Parameters. *M*. *cavernosa* population distribution parameters including site name, depth (m), geometric mean surface area (μ; cm^2^), skewness (g_1_), kurtosis (g_2_), standard deviation (SD), maximum colony surface area (95%; cm^2^), the probability that the populations is from a normal distribution (Pnorm), and the sample size (n) for each site surveyed and for all shallow sites and all mesophotic sites combined.

Site	Depth	*μ*	g_1_	g_2_	SD	95%	P_norm_	n
Rita/XL	10	2508	-0.638	0.497	0.834	16343	0.042	26
Coopers	10	1731	0.389	-0.369	0.518	8836	0.012	12
Spittal	10	1503	-0.535	-0.281	0.457	5655	0.006	21
Tuckers	10	1991	-1.904	4.283	0.737	5284	0.000	6
Devonshire	10	1808	-0.154	-1.161	0.555	7697	0.005	29
Hungry Bay	10	2145	0.046	-1.052	0.617	13586	0.010	31
Rita/XL	45	522	-0.755	0.992	0.687	7697	0.009	62
Coopers	45	330	-0.763	0.630	0.555	2389	0.010	66
Spittal	45	639	-0.304	-0.178	0.709	3927	0.043	36
Tuckers	45	349	-1.088	0.945	0.767	3041	0.031	58
Devonshire	45	322	-0.260	0.004	0.749	2513	0.152	96
Hungry Bay	45	441	-0.614	0.273	0.719	2513	0.078	108
**Shallow**	**10**	**1933**	**-0.499**	**0.452**	**0.620**	**16343**	**0.000**	**125**
**Deep**	**45**	**434**	**-0.627**	**0.369**	**0.713**	**7697**	**0.000**	**426**

### Colony abundance, surface area and percent cover

**Fig 2:**
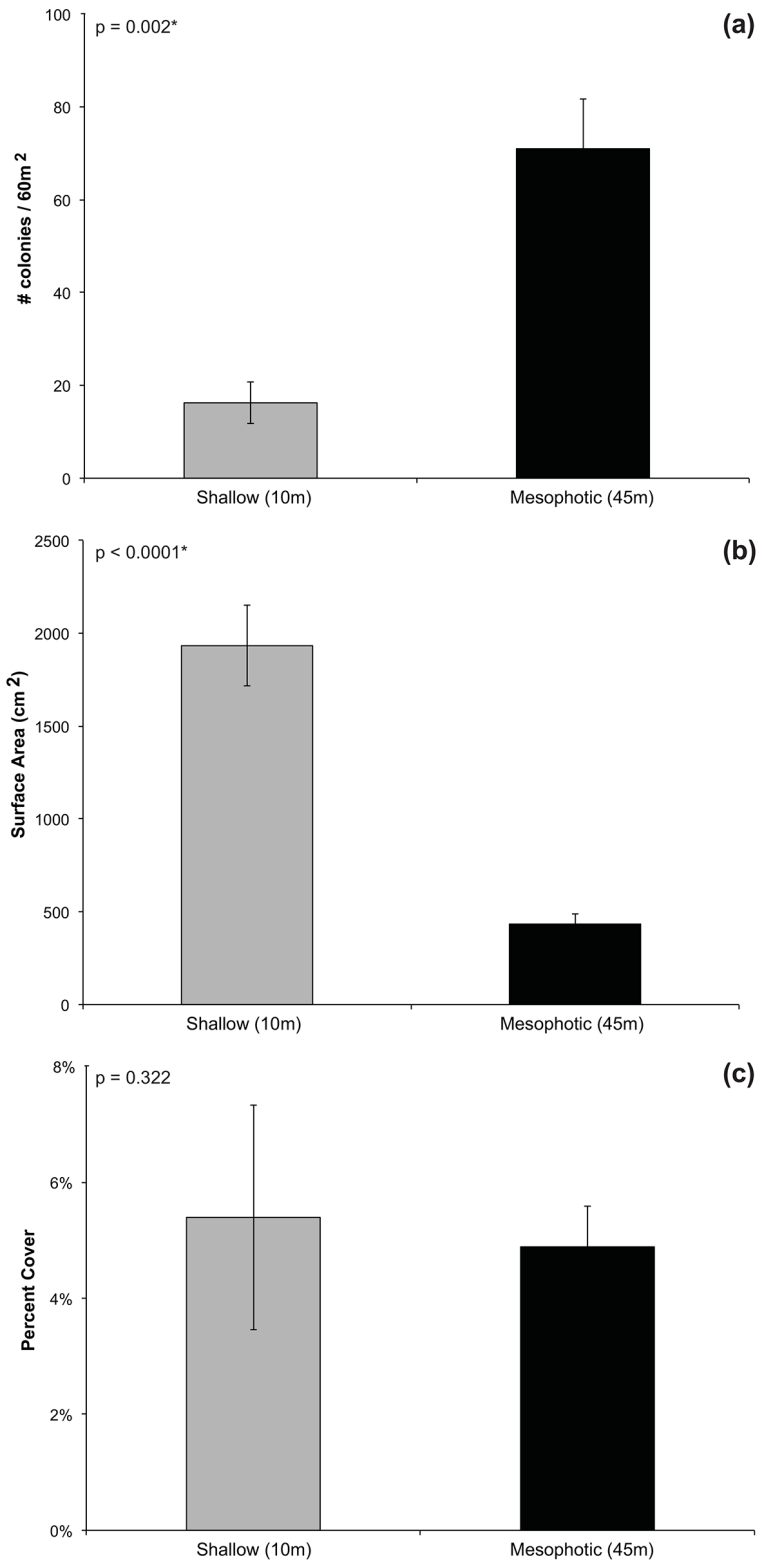
Colony Abundance, Surface Area, and Percent Cover by Depth. (a) mean number of *M*. *cavernosa* colonies per 60m^2^ ± SE at shallow (10m; gray bars) versus mesophotic (45m; black bars) sites (Rita/XL, Coopers, Tuckers, Spittal, Devonshire, Hungry Bay); (b) mean *M*. *cavernosa* colony surface area (cm^2^) ± SE at shallow (10m; gray bars) versus mesophotic (45m; black bars) sites; (c) mean percent cover ± SE of *M*. *cavernosa* at shallow (10m; gray bars) versus mesophotic (45m; black bars) sites (n = 6 per depth).

The mean density of colonies varied significantly by depth (p = 0.002, Students *t*-test, *F* = 0.106, n = 6), with higher colony density at mesophotic sites compared with shallow sites (Fig 2A). Colony surface area also varied significantly between depths (p<0.0001, Students *t*-test, *F* = 0.082, n = 6), where mean colony surface area was smaller at mesophotic sites compared with shallow sites (Fig 2B; Table 2). Mean colony surface area at shallow sites was typically 4.5 times greater than at deeper sites, and maximum surface area was 2.1 times greater at shallow sites (16343cm^2^) compared with mesophotic sites (7697cm^2^). This large discrepancy in individual colony surface area resulted in relatively equal contributions to mean percent cover at each depth (p = 0.322, Students *t*-test, *F* = 0.091, n = 6), despite the higher density of colonies at mesophotic sites (Fig 2C).

### Standard deviation, skewness and kurtosis

**Fig 3:**
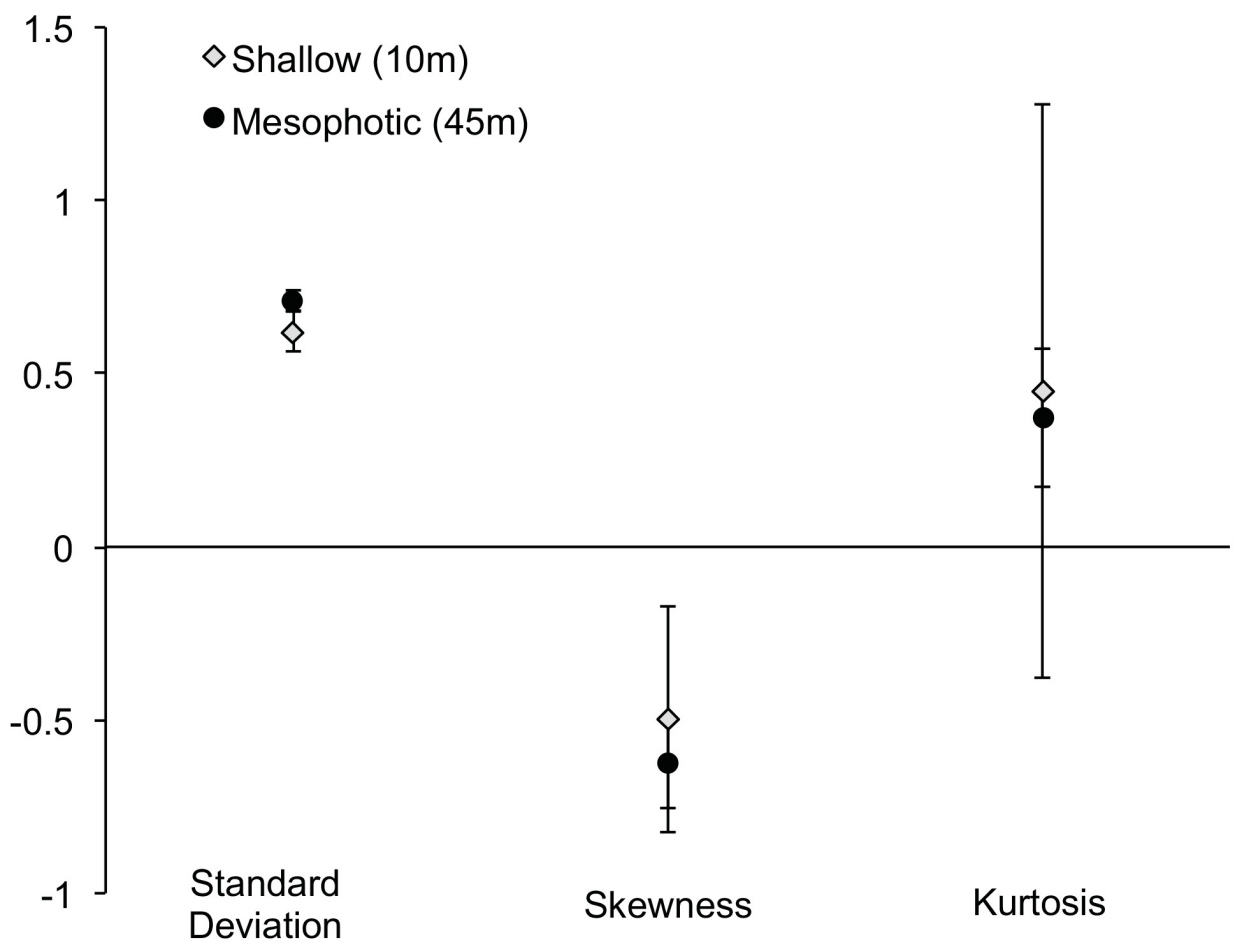
Distribution Parameters by Depth. Mean standard deviation, skewness, and kurtosis (± SE) of *M*. *cavernosa* population size-frequency distributions from measured colonies at shallow (10m; gray squares) and mesophotic (45m; black triangles) sites (Rita/XL, Coopers, Tuckers, Spittal, Devonshire, Hungry Bay).

Standard deviations of colony surface area data did not differ significantly between shallow and mesophotic sites (Fig 3; p = 0.262, Student’s *t*-test). This suggests that variation in colony surface area is similar at shallow and mesophotic sites.

The asymmetry around the mean of a size-frequency distribution is described as the skewness (g_1_; Table 2); where a negative g_1_ describes a distribution skewed to the left and a positive g_1_ distribution is skewed to the right. In a perfectly symmetrical distribution, g_1_ is zero [19]. Skewness did not vary significantly by depth (Fig 3; p = 0.649, Student’s *t*-test). Distributions at mesophotic and shallow sites were negatively skewed, indicating a lower frequency of colonies in the smaller size classes.

The degree of peakedness of a distribution around its central mean is described as kurtosis (g_2_), where a population can be either over centralized (leptokurtic, g_2_ > 0) or flatter than normal (platykurtic, g_2_ < 0). Kurtosis did not vary significantly by depth (Fig 3; p = 0.150, Mann-Whitney *U*-test), where the average kurtosis was 0.45 and 0.37 for shallow and mesophotic sites, respectively.

### Size-Frequency Distributions

**Fig 4:**
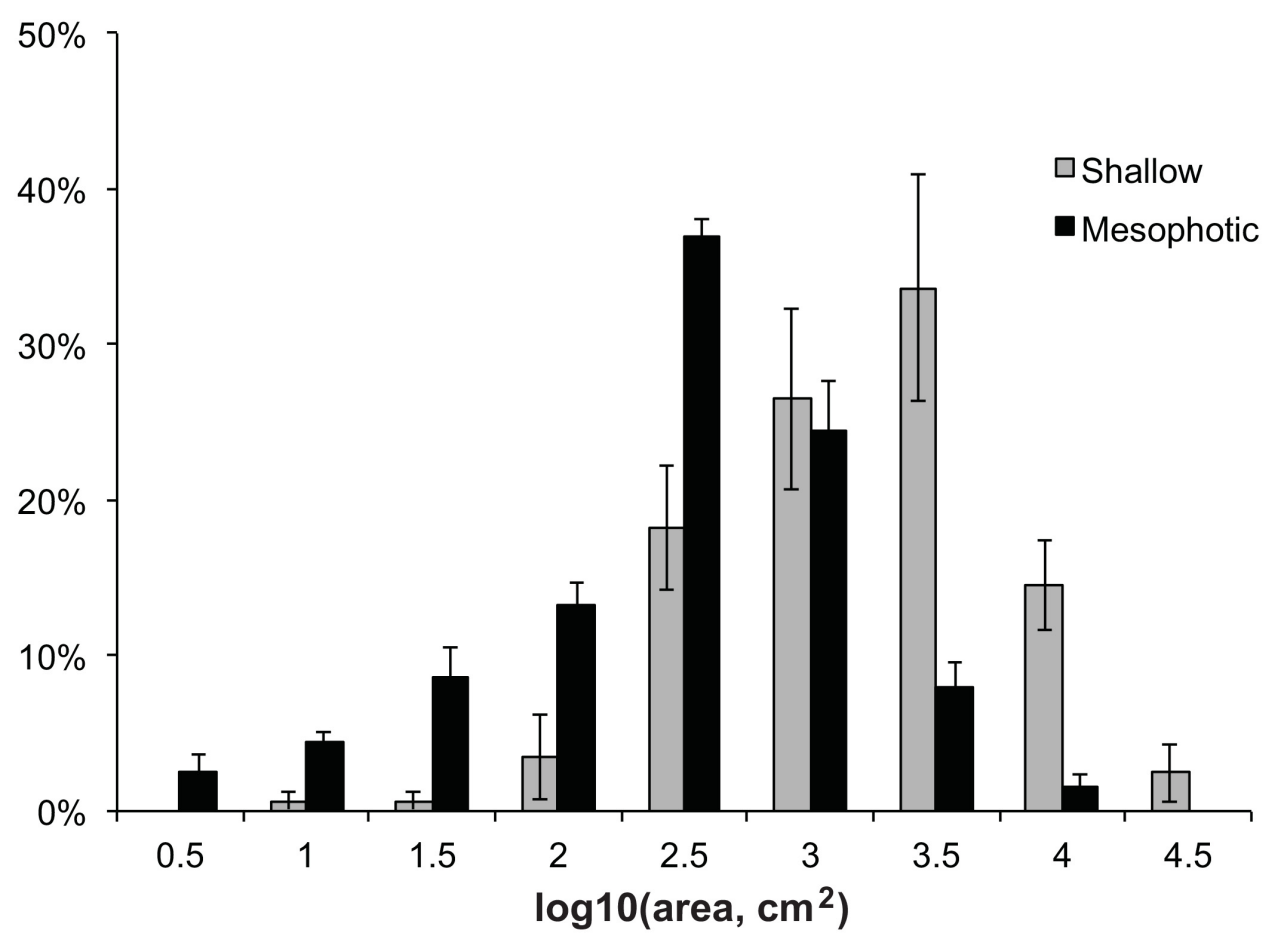
Mean Size-Frequency by Depth. Size-frequency distributions of *M*. *cavernosa* on a logarithmic scale represented as the mean proportion of individuals (± SE) within each log transformed size class for measured colonies from all shallow (10m; gray bars) and all mesophotic (45m; black bars) survey locations (Rita/XL, Coopers, Tuckers, Spittal, Devonshire, Hungry Bay).

Mean size-frequency distributions for shallow versus mesophotic sites are given in Fig 4. Logarithmically transforming colony surface area data greatly improved normality. Mean distribution patterns from shallow and mesophotic sites were bell-shaped, yet differed significantly from a normal distribution (Table 2; p<0.05, Shapiro-Wilk W test). Furthermore, mean distribution differed significantly between shallow versus mesophotic sites, being skewed towards larger colonies at shallow sites compared with mesophotic sites (Fig 4; p<0.001, Kolmogorov-Smirnov test).

**Fig 5:**
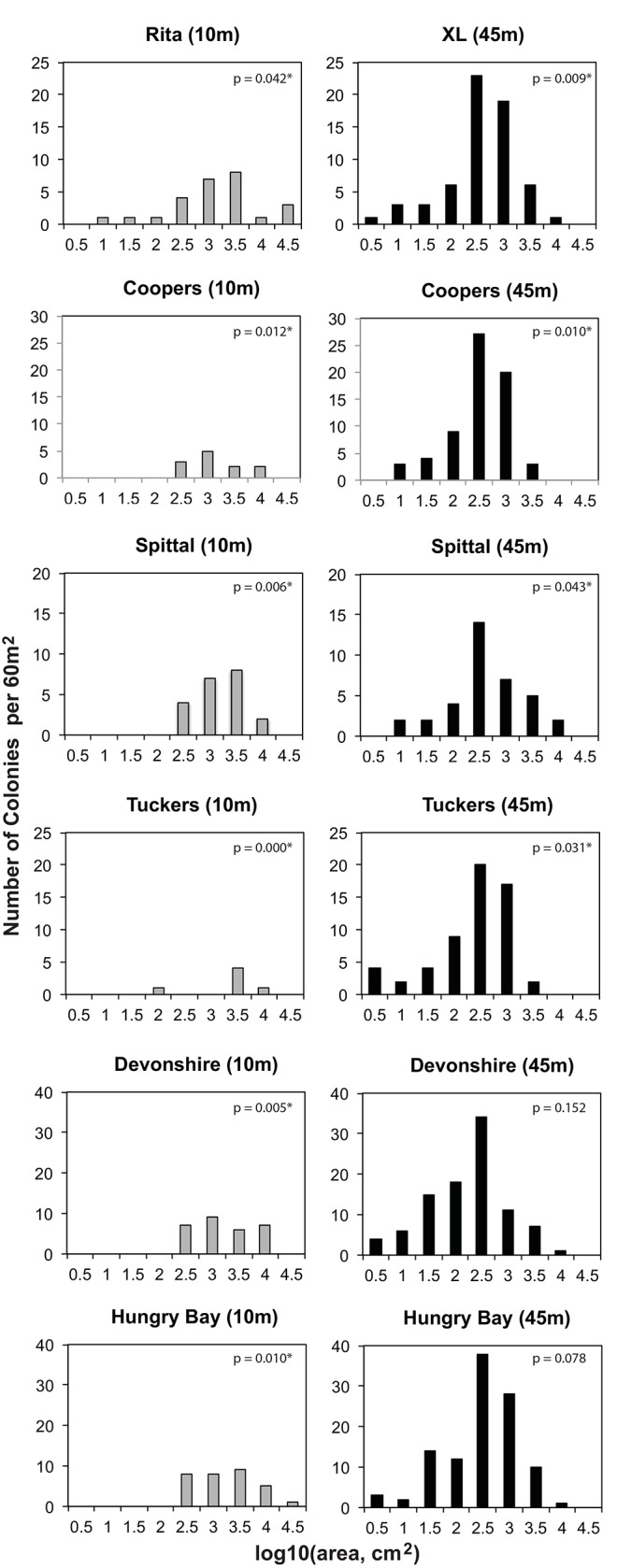
Size-Frequency by Site. Size-frequency distributions of *M*. *cavernosa* on a logarithmic scale represented as the number of individuals within each log transformed size class for colonies from each survey location (Rita/XL, Coopers, Tuckers, Spittal, Devonshire, Hungry Bay) at 10m (gray bars) and 45m (black bars) depths. Sites that differed significantly from a normal distribution are indicated with an asterisk (*; α<0.05).

**Fig 6:**
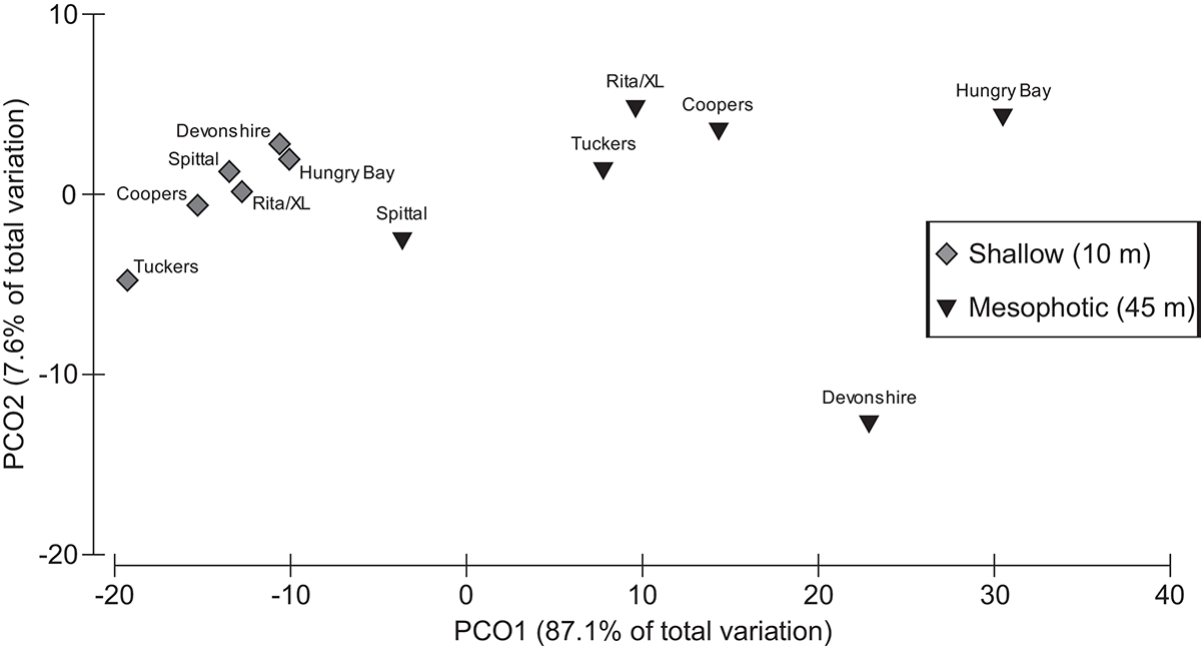
PCO of Population Structure by Site. Principal coordinates analysis (PCO) of *M*. *cavernosa* size-frequency distributions for each survey location (Rita/XL, Coopers, Tuckers, Spittal, Devonshire, Hungry Bay) at 10m and 45m depths. PCO1 and PCO2 axes together capture 94.7% of the total variation in size-frequency distribution.

Distributions within each of the mesophotic sites were bell-shaped, and 2 out of six sites did not differ from normal distribution (Table 2, Fig 5; p>0.05). Distributions within the shallow sites were more variable due to the lower density of individuals, with distributions at all sites differing from a normal distribution (Fig 5; p<0.05). Similarity of size-frequency distributions from each site were compared using the Spearman rank-correlation coefficient. These comparisons showed that distributions from the same depths (from distant sites) were more similar than those from adjacent sites at different depths (Table 3). The mean correlation coefficient of distributions from sites at the same depths was 0.29 (SD = 0.08, n = 30), while the mean correlation coefficient of comparisons from adjacent sites at different depths was 0.24 (SD = 0.28, n = 6). These means are significantly different (p = 0.006, Mann-Whitney *U*-test). The high degree of similarity between distributions from the same depth suggests that the population structure of *M*. *cavernosa* has depth specific characteristics. The PCO results are provided in Fig 6, confirming a clear separation of the size-frequency distributions between depths and more similarity among sites of the same depth than between paired sites at different depths.

**Table 3:** Correlation Coefficients. Spearman rank correlation coefficient values for comparisons of size-frequency distributions of *M*. *cavernosa* between sites. Values above the staggered line are comparisons among shallow sites; values below the staggered line are comparisons among mesophotic sites; values between the staggered lines are between adjacent shallow and mesophotic sites. Significant correlations (statistically similar; α = 0.05) are indicated in bold.

Spearman rank-correlation coefficient						
	Rita/XL	Coopers	Spittal	Tuckers	Devonshire	HungryBay
Rita/XL	**0.641**	**0.717**	0.279	**0.795**	**0.653**	**0.873**
Coopers	**0.934**	0.504	0.203	**0.934**	**0.988**	**0.880**
Spittal	**0.962**	**0.881**	**0.756**	0.418	0.229	0.306
Tuckers	**0.856**	**0.879**	**0.715**	-0.160	**0.903**	**0.943**
Devonshire	**0.905**	**0.931**	**0.816**	**0.892**	0.296	**0.847**
HungryBay	**0.917**	**0.919**	**0.829**	**0.941**	**0.939**	0.332

### Nutrients and Seawater Temperature

**Fig 7:**
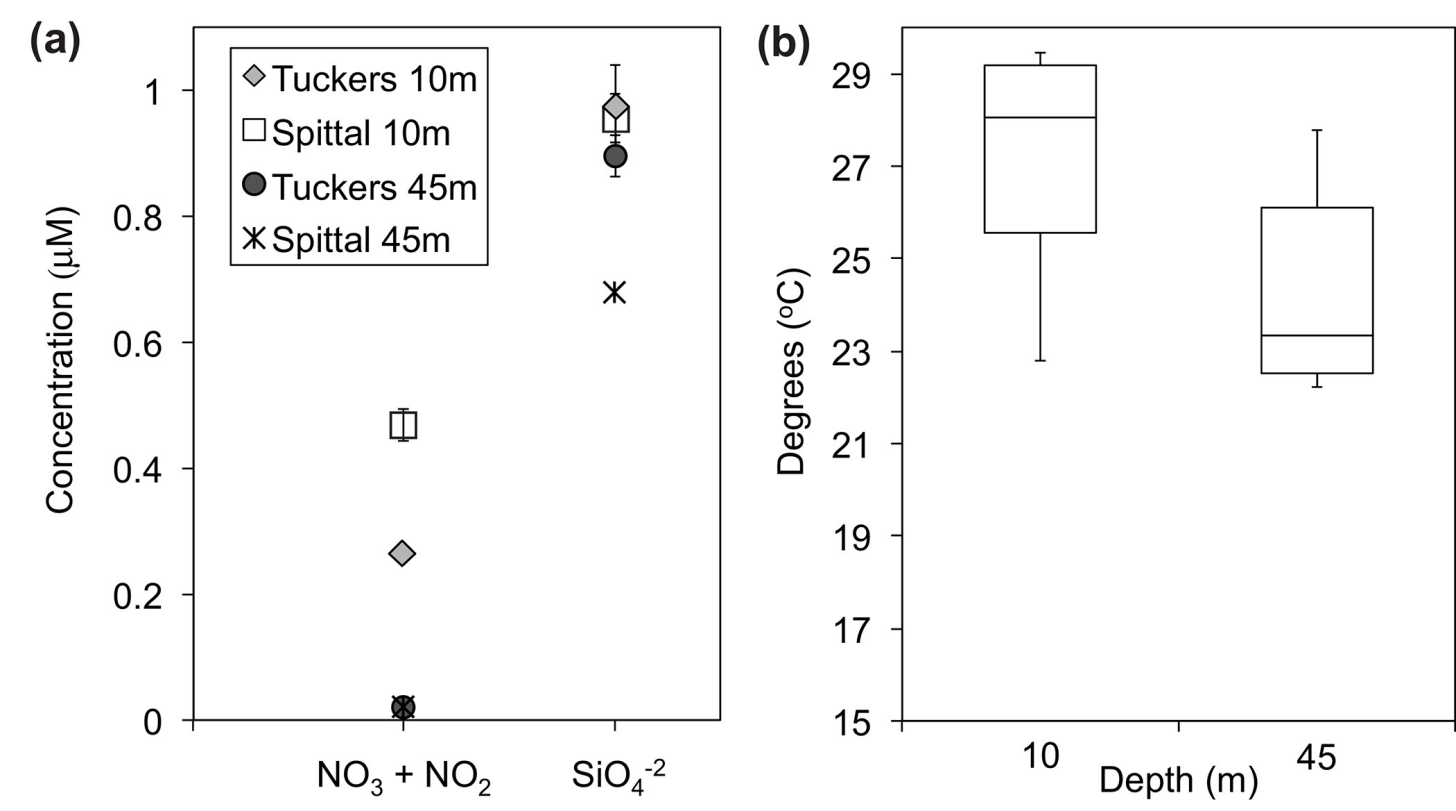
Nutrient Concentration and Temperature by Depth. (a) mean (±SD) concentration (μM) of nitrate (NO_3_) + nitrite (NO_2_) and silicate (SiO_4_^-2^) on shallow (10m; n = 4 per site) versus mesophotic sites (45m; n = 4 per site) from water samples collected September 5, 2015 (NO_3_ + NO_2_, p<0.0001, Tuckers, p = 0.019, Spittal; SiO_4_^-2^, p = 0.001, Tuckers, p = 0.058, Spittal; Student’s *t*-tests); (b) box blot of seawater temperature at shallow (10m) and mesophotic (45m) sites showing median values (solid horizontal line), 25^th^ and 75^th^ percentile values (box outline), and minimum and maximum values (whiskers) recorded between July 2014 and January 2015 from 6 paired shallow (10m) and mesophotic (45m) survey sites (2 dives per site); Rita/XL, Coopers, Tuckers, Spittal, Devonshire, and Hungry Bay (p<0.0001, Students *t*-test, n = 6).

Nutrient concentrations were higher on shallow sites compared with mesophotic sites (Fig 7A), with significant differences found in concentrations of nitrate and nitrite between depths (p<0.0001, Tuckers; p = 0.019, Spittal; Student’s *t*-tests, n = 4) and silicate between depths at Tuckers (p = 0.001, Student’s *t*-test, n = 4), but not at Spittal (p = 0.058, Student’s *t*-test, n = 4). Mean seawater temperatures also differed significantly by depth, being higher on shallow sites compared with mesophotic sites (p<0.0001, Student’s *t*-test, n = 6). Likewise, variation in temperature was more pronounced on shallow sites, ranging from 22.8 to 29.5°C, compared with mesophotic sites, ranging from 22.2 to 27.8°C (Fig 7B).

## Discussion

This study documents the population structure of *M*. *cavernosa* at mesophotic versus shallow reefs in Bermuda and reveals depth specific characteristics of these populations. Our analyses show that size-frequency distributions of populations at shallow reefs vary significantly from those at mesophotic reefs (Fig 4), with colonies from neighboring reefs at the same depths being more similar to one another than to those from adjacent populations at different depths (Figs 4 and 5, Table 3). These results suggest that conditions that vary with depth, such as light, seawater temperature, and nutrient concentration, likely influence *M*. *cavernosa* population structure. Overall, this study found the distribution of populations at the mesophotic reef sites examined on Bermuda’s south shore is shifted towards smaller individuals relative to shallow reefs (Fig 4). Likewise, the average colony surface area at these mesophotic reefs was significantly smaller than at shallow reefs (Fig 2A). However, it is important to note that a large degree of variation in colony morphology was observed at the different depths, with colonies at mesophotic sites being predominately flat and disc shaped, compared with colonies at shallow sites that varied from flat, to encrusting, to massive boulders. Thus, using diameter to estimate surface area likely underestimated total surface area of shallow-water colonies, resulting in a conservative estimate of colony size differential by depth in this study. These data indicate, therefore, that growth and maximum colony surface area may be limited on mesophotic reefs and suggests that maximum *M*. *cavernosa* colony surface area is likely controlled by environmental conditions that may limit energetic resources, such as light and nutrient availability.

The smaller surface areas of *M*. *cavernosa* colonies found at mesophotic reefs in this study conforms to previous studies that document a decrease in coral colony surface area with depth [35] and the predominance of small colonies at deeper depth distributions [36, 37]. Smaller colony surface area may be a result of nutrient limitation at mesophotic reefs as nutrient analyses of adjacent mesophotic and shallow reefs in this study indicate that nutrients are significantly reduced at these mesophotic sites compared with shallow sites (Fig 7A). Likewise, Lesser et al. [25] document a reduction in phytoplankton availability and marked decreases in light-dependent productivity with depth, indicating that energy required for calcification and growth may indeed be limited for mesophotic corals [38, 39]. Under low-light and nutrient-limited conditions such as those present at mesophotic reefs, corals decrease metabolic demand through reduced respiration [40], slower growth, and morphological adaptations. For example, Grigg [41] found skeletal extension rates of *Porites lobata* declined exponentially with PAR from 3 to 50m in Hawaii. Likewise, Fricke et al. [35] report skeletal extension rates in *Leptoseris fragilis* at 90 to 120m of 0.5–0.8mm year^-1^, which is significantly lower than typical rates reported for other non-branching shallow water corals ranging from 1.0–8.5mm year^-1^ [42]. Alternatively, Bongaerts et al. [43] report an average growth rate of 22.0mm year^-1^ for *Agaricia grahamae* fragments transplanted to 60m in Curacao, which is similar to growth rates in the congeneric species *A*. *humilis* and *A*. *agaricites* from shallow reefs (<30m) in the same region [44, 45]. Additionally, metabolic demands may be met through increased reliance on heterotrophy in conditions where primary production is limited. In the Bahamas, Lesser et al. [25] document a transition from autotrophy to heterotrophy with depth in populations of *M*. *cavernosa* between 45 and 61m associated with significant declines in primary productivity. Whether the energy consumed through heterotrophy is substantial enough to compensate for the reduction in primary production and maintain metabolic rates similar to shallow corals, however, is unclear. Thus, future studies of *M*. *cavernosa* on mesophotic reefs should include examinations of skeletal extension rates to determine rates of growth.

Despite the smaller surface area per colony of *M*. *cavernosa* at mesophotic sites, the relatively high density resulted in equal contributions to percent cover at mesophotic and shallow reefs (Fig 2). The high density of *M*. *cavernosa* colonies at mesophotic reefs may be related to lack of competition with other coral species that are unable to adapt to conditions at this depth. Diversity decreases dramatically at mesophotic reefs with only a handful of scleractinian species known to reside at these depths in Bermuda, including *A*. *fragilis*, *M*. *carambi*, *M*. *decactis*, *M*. *cavernosa*, *O*. *franksii*, *P*. *porites*, *S*. *michelini*, and *S*. *cubensis*. Among them, *M*. *cavernosa* is the most predominant and is the only coral documented to survive in Bermuda below 70m [21, 46]. On surveys conducted for this study, *M*. *cavernosa* was the most abundant scleractinian species at these mesophotic sites, while *A*. *fragilis* was the second most abundant species with low rates of occurrence (Goodbody-Gringley, pers. obs.). Furthermore, recruitment for most taxa is documented to decline dramatically below 50m, indicating that competitive exclusion has less influence on community structure at depth [47]. Thus, competition for space is likely not a limiting factor for population density on mesophotic reefs, as restricted light and nutrient availability reduces the abundance and diversity of competitive species allowing *M*. *cavernosa* to become well established [47, 48].

Species distribution and population structure are highly influenced by characteristics of the physical environment such as temperature and wave energy, which vary with depth and thus affect coral population dynamics on mesophotic reefs. Thermally induced coral bleaching is known to cause significant mortality on shallow-water reefs [49], however mesophotic corals appear to be well insulated from the effects of increased sea surface temperature (SST), which may be in part due to the lower degree of variability in SST experienced on mesophotic reefs compared with shallow reefs (Fig 7B) [8, 50, 51]. Although increased SST is a reliable indicator of increasing temperature at mesophotic depths >30m [52], Lesser and Slattery [51] report bleaching to be virtually absent on corals inhabiting mesophotic reefs. Reduced occurrences of bleaching events on mesophotic reefs is likely due to a lower maximum SST (Fig 7B) and solar isolation [53] at depth, despite the potential for cold-water stress to induce bleaching as reported elsewhere on Caribbean reefs [54–56]. Likewise, hydrodynamic disturbance and exposure to wave energy are major factors influencing community structure in shallow reef systems. Such disturbances are minimal on mesophotic reefs, however, as surface wave energy attenuates with depth [57, 58]. Corals inhabiting mesophotic reefs are, therefore, buffered from direct physical damage from rough hydrodynamic conditions, which may contribute to the long-term stability of these ecosystems, although episodic storm events may cause fragmentation of branching, foliose and columnar coral colonies at depth [9, 59]. Additionally, human-mediated stresses appear to be reduced on mesophotic reefs due primarily to increased distance from human populations and greater depths than nearby shallow reef systems [60]. Therefore, corals inhabiting mesophotic zones may be protected from biotic and abiotic impacts that typically occur on shallow-water coral reefs.

In fact, the results of this study indicate that colonies of *M*. *cavernosa* appear to form relatively stable populations on mesophotic reefs in Bermuda. Mean population size structure was bell-curved (Fig 4), and standard deviation, skewness, and kurtosis did not vary greatly by site (Table 2). These results suggest that the mesophotic zone, which extends around the perimeter of the Bermuda platform, creates a viable habitat able to support an established population of *M*. *cavernosa*.

Likewise, the mean size-frequency distribution of shallow reef populations was also bell curved, however, the overall size structure of shallow sites was shifted towards larger individuals with the smallest size classes underrepresented (Fig 4). Previous studies on coral population structures suggest that environmental deterioration may skew populations towards a greater proportion of larger individuals [13, 19]. While the results of the present study may indicate that the shallow reef environment in Bermuda is less stable than mesophotic regions, there was no statistical difference in skewness of the populations preventing any conclusive remarks as to the stability of shallow sites versus mesophotic sites.

These findings support previous survey work conducted with submersibles and ROV’s in other regions that show stable populations of scleractinian corals on mesophotic reefs, which have not undergone declines similar to those seen on their shallow water counterparts [6]. This apparent stability has led to the development of the “Deep Reef Refugia Hypothesis”, which posits that coral populations at depths greater than 30m could serve as a source/sink for genetic diversity and future repopulation of shallow regions [9]. Several recent studies have undertaken comparisons of conspecifics at neighboring deep and shallow reefs, and show that while a slight degree of genetic discontinuity appears to be present at certain locations, other shallow/deep populations display evidence of genetic connectivity, supporting the possibility of repopulation of deteriorating shallow reefs by deep reef populations [61–63]. Understanding the degree of genetic connectivity among shallow and mesophotic corals will, therefore, ultimately indicate the ability of deep reefs to contribute to shallow reef resilience. Likewise, determining the health and stability of mesophotic coral populations through demographic analyses will suggest the viability of these reefs to serve as a source of propagules to maintain shallow water reefs and help guide future management and conservation strategies [25].

The results presented here represent a baseline assessment of coral population structure and reef condition on MCE’s in Bermuda. As the technology of mixed-gas closed circuit diving advances, it is anticipated that research on MCE’s will rapidly increase. Access to baseline data on community structure and reef condition will be imperative for future examinations of population demography, assessments of connectivity, projections of ecosystem change, and the overall resilience of global coral reef systems.
